# 

**Published:** 1894-04-14

**Authors:** 


CONTENTS.

**Ubi est Morbus?** - - - - - - - - - - - 23

**Around the Hospitals** - - - - - - - - - - - 24

**Modern Medico-Psychology and Psychiatry. III.** - - 25

**Famous Poisoners in Fiction. IX.** - - - - - - - 26

**Modern Views of Malaria. I.** - - - - - - - - 27

**The First Century of English Medical Literature. I.** - 28

**Medical Progress and Hospital Clinics—**

Electricity and Its Use in Medicine. II. - - - - - - 31

Dilatation of the Stomach - - - - - - - - - 33

Medical Progress—Pulmonary Tuberculosis - - - - 35

Progress in Surgery—Malignant Growths—Loretine - - 36

New Appliances and Things Medical - - - - - - 37

**Annotations.**—The Scientific Method—The Chelsea Hospital and Dr. Parkes—Should Journalists Marry? - - - - - - 29

**Notes and News** - - - - - - - - - - - - 30

**The International Medical Congress at Rome** - - - 38

**The Institutional Workshop—**

Hospital Finance. V.—Patients' Payments - - - - - 39

Asylums in India. V.—The Punjaub - - - - - - - 40

Practical Departments - - - - - - - - - - 40

Hospital Festivals, Meetings, &c. - - - - - - - 41

Construction Notes - - - - - - - - - - 42

Practical Points - - - - - - - - - - - 42

**Scraps and Gleanings** - - - - - - - - - 42

EXTRA SUPPLEMENT.—THE NURSING MIRROR.

**News from the Nursing World.**—Where to Save—Useful Lives—Old Linen—A Successful Bazaar—Where Was the Doctor?—Gloucester—A Wise Step—Proposed Training at Chelmsford—Sectarian Charities—Convalescents at Teeside—The Carna Hospital—An Appeal from India—A Musical Treat—Tricycles for District Nursing—Short Items - - - - - - xi

**On General Nursing. VII.**—Catarrh (*continued*) - - - - xiii

**A Nursing Home at Kew** - - - - - - - - - xiv

**The Ethics of Nursing. I.** - - - - - - - - - xiv

**The Nursing of Diphtheritic Paralysis** - - - - - xv

**Novelties for Nurses** - - - - - - - - - - xv

**Nurses' Friends and Foes** - - - - - - - - - xvi

**The North-Eastern Hospital for Children** - - - - - xix

**Notes and Queries** - - - - - - - - - - - xix

**Our Examinations** - - - - - - - - - - - xix

**Where to Go** - - - - - - - - - - - - xix

**Minor Appointments** - - - - - - - - - - xviii

**A Generous Bequest to Nurses** - - - - - - - - xix

**Everybody's Opinion** - - - - - - - - - - xx

**For Reading to the Sick** - - - - - - - - - xx

